# Copy number variation of horse Y chromosome genes in normal equine populations and in horses with abnormal sex development and subfertility: relationship of copy number variations with Y haplogroups

**DOI:** 10.1093/g3journal/jkac278

**Published:** 2022-10-13

**Authors:** Caitlin Castaneda, Lara Radović, Sabine Felkel, Rytis Juras, Brian W Davis, Ernest Gus Cothran, Barbara Wallner, Terje Raudsepp

**Affiliations:** Department of Veterinary Integrative Biosciences, School of Veterinary Medicine and Biomedical Sciences, Texas A&M University, College Station, TX 7784-4458, USA; Department of Biomedical Sciences, Institute of Animal Breeding and Genetics, University of Veterinary Medicine Vienna, Vienna 1210, Austria; Vienna Graduate School of Population Genetics, University of Veterinary Medicine Vienna, Vienna 1210, Austria; Department of Biomedical Sciences, Institute of Animal Breeding and Genetics, University of Veterinary Medicine Vienna, Vienna 1210, Austria; Vienna Graduate School of Population Genetics, University of Veterinary Medicine Vienna, Vienna 1210, Austria; Department of Biotechnology, Institute of Computational Biology, BOKU University of Life Sciences and Natural Resources, Vienna 1190, Austria; Department of Veterinary Integrative Biosciences, School of Veterinary Medicine and Biomedical Sciences, Texas A&M University, College Station, TX 7784-4458, USA; Department of Veterinary Integrative Biosciences, School of Veterinary Medicine and Biomedical Sciences, Texas A&M University, College Station, TX 7784-4458, USA; Department of Veterinary Integrative Biosciences, School of Veterinary Medicine and Biomedical Sciences, Texas A&M University, College Station, TX 7784-4458, USA; Department of Biomedical Sciences, Institute of Animal Breeding and Genetics, University of Veterinary Medicine Vienna, Vienna 1210, Austria; Department of Veterinary Integrative Biosciences, School of Veterinary Medicine and Biomedical Sciences, Texas A&M University, College Station, TX 7784-4458, USA

**Keywords:** male-specific Y, multicopy, ampliconic, male fertility, horse breeds, patrilines, SCNT, cryptorchidism, haplotype

## Abstract

Structural rearrangements like copy number variations in the male-specific Y chromosome have been associated with male fertility phenotypes in human and mouse but have been sparsely studied in other mammalian species. Here, we designed digital droplet PCR assays for 7 horse male-specific Y chromosome multicopy genes and *SRY* and evaluated their absolute copy numbers in 209 normal male horses of 22 breeds, 73 XY horses with disorders of sex development and/or infertility, 5 Przewalski’s horses and 2 kulans. This established baseline copy number for these genes in horses. The *TSPY* gene showed the highest copy number and was the most copy number variable between individuals and breeds. *SRY* was a single-copy gene in most horses but had 2–3 copies in some indigenous breeds. Since *SRY* is flanked by 2 copies of *RBMY*, their copy number variations were interrelated and may lead to *SRY*-negative XY disorders of sex development. The Przewalski’s horse and kulan had 1 copy of *SRY* and *RBMY*. *TSPY* and *ETSTY2* showed significant copy number variations between cryptorchid and normal males (*P < *0.05). No significant copy number variations were observed in subfertile/infertile males. Notably, copy number of *TSPY* and *ETSTY5* differed between successive male generations and between cloned horses, indicating germline and somatic mechanisms for copy number variations. We observed no correlation between male-specific Y chromosome gene copy number variations and male-specific Y chromosome haplotypes. We conclude that the ampliconic male-specific Y chromosome reference assembly has deficiencies and further studies with an improved male-specific Y chromosome assembly are needed to determine selective constraints over horse male-specific Y chromosome gene copy number and their relation to stallion reproduction and male biology.

## Teaser

This manuscript presents digital droplet PCR-based copy number analysis of horse Y chromosome multicopy genes in normal multi-breed equine populations including patrilines and in horses with developmental or reproductive disorders. The authors compare the dynamics of copy number variation of Y-linked genes with SNP-based horse Y haplotypes and the findings are discussed in the context of stallion reproduction and male biology.

## Introduction

The Y chromosome is one of the most structurally, functionally, and evolutionarily distinct regions in the mammalian genome. During the evolution of the eutherian sex chromosomes from the same autosomal ancestor, the Y acquired a dominant testis-determining locus, which led to gradual cessation of X–Y recombination (Lahn and Page 1999; Bellot *et al.* 2014; Waters and Ruiz-Herrera 2020). Reduced X–Y recombination initiated a cascade of other evolutionary events in the Y such as an increase of structural rearrangements (inversions), gradual loss of ancestral genes, and reduction in size (Graves 2006; Bellot *et al.* 2014; Hughes *et al.* 2015, 2020). On the other hand, the lack of recombination and male-specific transmission, favored the acquisition, and expansion of male-benefit genes in the Y, several of which became multicopy or ampliconic with high (>99%) sequence identity between the copies (Skaletsky *et al.* 2003; Hughes *et al.* 2020). The presence of high identity euchromatic repeats has made Y chromosome sequence assembly challenging. Only 4 species have finished Y assemblies: human (Skaletsky *et al.* 2003), chimp (Hughes *et al.* 2010), rhesus macaque (Hughes *et al.* 2012, 2015), and mouse (Soh *et al.* 2014). As well, draft Y assemblies are available for several species, including domestic animals such as cat and dog (Li *et al.* 2013; Brashear *et al.* 2018), pig (Skinner *et al.* 2016), cattle (Bellot *et al.* 2014; Hughes *et al.* 2020), horse (Janečka *et al.* 2018), goat (Xiao *et al.* 2021), and sheep (Li et al. 2021).

The increasing number of high-quality Y assemblies expands the scope of comparative studies across eutherian species [see, for example, Li *et al.* 2013; Bellot *et al.* 2014; Cortez *et al.* 2014; Janečka *et al.* 2018; Martinez-Pacheco *et al.* 2020), but also allows the study of intraspecific Y sequence variation. Of particular interest are copy number variations (CNVs) of multicopy and ampliconic genes. High sequence identity between the copies makes those genes prone to nonallelic homologous recombination resulting in deletions and duplications (Vogt *et al.* 1996; Repping *et al.* 2006; Lange *et al.* 2009).

Male-specific Y (MSY) CNVs have been studied for decades in humans and some were shown to have a direct link to male fertility. Various microdeletions have been described to affect the efficiency of spermatogenesis causing azoospermia, oligozoospermia, or oligoasthenozoospermia in men (O'Brien *et al.* 2010). Those CNVs are represented by 3 critical regions in the human MSY, known as Azoospermia Factors (AZF) AZFa, AZFb, and AZFc (Reijo *et al.* 1996; Saxena *et al.* 1996; O'Brien *et al.* 2010) which harbor several single- and multicopy genes that are essential for sperm development. The severity of a subfertility phenotype in each case depends on the size of the deletion in addition to the exact region where it is located. Even though the organization and gene content of the Y chromosome is different across eutherians (Martinez-Pacheco *et al.* 2020), similar association between MSY CNVs and male fertility has been observed in other species. For example, deletions in the ampliconic long arm of the mouse Y chromosome lead to decreased sperm quality and infertility (Ellis *et al.* 2005; Toure *et al.* 2005; Grzmil *et al.* 2007). Studies of MSY CNVs, with particular focus on amplicon variation, have also been initiated in primates (Ghenu *et al.* 2016; Oetjens *et al.* 2016; Tomaszkiewicz *et al.* 2016; Vegesna *et al.* 2020), murines (Morgan and Pardo-Manuel de Villena 2017), groups of bovids (Mukherjee *et al.* 2013, 2015; Oluwole *et al.* 2017; Pei *et al.* 2019; Zhang *et al.* 2019), dogs (Krzeminska *et al.* 2022), felids (Brashear *et al.* 2018), and the donkey (Han *et al.* 2017). Though, aside from men and mice, there is limited knowledge about the association between MSY CNVs and male fertility and/or sex development in other species. In cattle, it is suggested that a lower number of *TSPY* copies could affect semen quality (Mukherjee *et al.* 2015). In dogs, with 7 copies of the *SRY* gene (Li *et al.* 2013), reduced *SRY* CN is thought to be associated with an increased risk of disorders of sex development (DSD) (Krzeminska *et al.* 2022). Despite the economic importance of stallion fertility, CNV studies have not yet been conducted for the 15 multicopy genes (Janečka *et al.* 2018) in horse MSY. However, the novel acquisition and amplification of these genes in MSY, and testis-specific transcription suggest a role in male reproduction (Paria *et al.* 2011; Janečka *et al.* 2018).

Another important form of MSY intraspecific variation are single nucleotide variants (SNVs) which are excellent markers for determining Y haplotypes (HTs) and tracing the history of patrilines. MSY HT data have been widely used to infer the phylogenetic history of human patrilineages worldwide (Jobling and Tyler-Smith 2017; Grugni *et al.* 2019; Bisso-Machado and Fagundes 2021; Ilumäe *et al.* 2021; Navarro-Lopez *et al.* 2021; Martiniano *et al.* 2022) but also for the study of paternal ancestry of populations in domestic species such as cattle (Edwards *et al.* 2011; Xia *et al.* 2019; Escouflaire and Capitan 2021), dogs (Ding *et al.* 2012; Oetjens *et al.* 2018), goats (Vidal *et al.* 2017), and pigs (Guirao-Rico *et al.* 2018; Ai *et al.* 2021). Despite the unprecedented low nucleotide variation of horse MSY (Wallner *et al.* 2017; Wutke *et al.* 2018), HT data for horses have significantly expanded over the last decade (Wallner *et al.* 2017; Castaneda *et al.* 2019; Felkel *et al.* 2019; Liu *et al.* 2020) including a stable MSY phylogeny built on SNVs (Felkel *et al.* 2019). However, only a few studies in human and primates have integrated MSY CNV and HT data (Ye *et al.* 2018) to determine whether there is any correlation between the 2 forms of variation (Vegesna *et al.* 2019).

In this study, we develop accurate copy number (CN) evaluation assays for horse MSY multicopy genes and determine the range of MSY CNVs in a multibreed equine population sample and some wild equids. Once the equine MSY “natural variation” is established, we compare MSY CNV patterns with MSY HTs for correlation. Finally, we evaluate MSY gene CNs in a group of subfertile males and horses with DSD to identify CNVs associated with the phenotypes.

## Materials and methods

### Ethics statement

Procurement of samples followed the United States Government Principles for the Utilization and Care of Vertebrate Animals Used in Testing, Research and Training. These protocols were approved as AUP and CRRC #2018-0342 CA at Texas A&M University.

### Animals, samples, and phenotypes

Genomic DNA (gDNA) samples of 289 male horses and equids were available from the repositories of Molecular Cytogenetics and Animal Genetics Laboratories at Texas A&M University and Institute of Animal Breeding and Genetics at the Veterinary University of Vienna. gDNA was extracted from hair follicles or peripheral blood with a Gentra Puregene Tissue or Blood kit (Qiagen), respectively, following the manufacturer’s protocols. DNA quality and quantity were evaluated with Nanodrop ND-8000 spectrophotometer (ThermoFisher) and Qubit 4.0 fluorometer (ThermoFisher). Additional evidence of the quality of these gDNA samples was the fact that most have been successfully used for recently published research in equine population genetics (Wallner *et al.* 2017; Castaneda *et al.* 2019; Felkel *et al.* 2019) and clinical genomics (Raudsepp *et al.* 2010; Ghosh, Davis, *et al.* 2020; Raudsepp 2020).

The samples included a normal control cohort of 216 male equids: 209 domestic horses (*Equus caballus*) of 22 breeds or breed mixes, 5 Przewalski's horses (*Equus caballus przewalskii*), and 2 kulans (*Equus hemionus kulan*) (Supplementary Table 1). The cohort of normal male horses also included a Thoroughbred stallion *Bravo—*the DNA donor for the horse MSY assembly (Janečka *et al.* 2018) and the control male for all droplet digital PCR (ddPCR) experiments in this study. A horse was considered normal if there was no information suggesting otherwise. Because only a small number of male horses are used for breeding, evidence of fertility was available for only a few individuals. Additionally, we used gDNA from 73 abnormal male horses: 24 cryptorchid (CO) American Quarter Horses (AQHs) (6 bilateral, bi-CO; 18 unilateral, uni-CO), 29 horses of ambiguous sex with confirmed *SRY*-positive or *SRY*-negative 64, XY DSD, and 12 males with heterogeneous subfertility phenotypes. All horses in the abnormal group originated from the depository of Molecular Cytogenetics laboratory as subjects for chromosome analysis. Their phenotypes have been characterized by referring veterinarians *(*see Bugno-Poniewierska and Raudsepp 2021), these horses have been tested for the *SRY* gene by PCR and karyotyped following the standard procedures in equine clinical cytogenetics (Ghosh, Carden, *et al.* 2020). We also used gDNA from related male horses: 9 males from 4-, 2-, or 3-generation families and 8 males within 2 families produced by somatic cell nuclear transfer (SCNT). Detailed information about the abnormal horses and SCNT-horses is presented in Supplementary Tables 1 and 6–8.

### ddPCR analysis

Sequences of all 15 horse MSY multicopy genes (Janečka *et al.* 2018) were bioinformatically inspected for suitability for ddPCR following assay requirements (Digital Droplet PCR Application Guide, BioRad: https://www.bio-rad.com/webroot/web/pdf/lsr/literature/Bulletin_6407.pdf; last accessed February 2022) and MSY sequence properties. Assays were successfully designed and optimized for 7 horse MSY multicopy genes, single-copy *SRY*, and autosomal control genes *MYOZ1* and/or *MSTN* (Supplementary Table 3). Primers were designed with Primer3 v.0.4.0 software (Untergasser *et al.* 2012) using reference sequences for horse MSY (Janečka *et al.* 2018) and EquCab3 (Kalbfleisch *et al.* 2018) so that the size of PCR products was in the range of 75–200 base pairs. Fluorescently labeled (FAM for MSY genes, VIC for autosomal *MYOZ1*, and *MSTN*) hydrolysis probes (TaqMan) were designed with PrimerQuest tool (Integrated DNA Technologies: https://www.idtdna.com/pages/tools/primerquest?utm_source=google&utm_medium=cpc&utm_campaign=ga_primerquest&utm_content=ad_group_primerquest&gclid=CjwKCAjwj8eJBhA5EiwAg3z0m61WFuiwsyllkEVNf0aRgPZDO_d_q-nYJoaYanXJLKSupBjann3cJxoCa3kQAvD_BwE; last accessed February 2022). ddPCR reactions were conducted as previously described (Castaneda *et al.* 2021). The template gDNA was cleaved with EcoRI (10 U/µl; Invitrogen) or NspI (10 U/µl; New England Biolabs) restriction enzymes into <5 kb fragments to fit into individual droplets. The restriction enzyme chosen for the experiment was dependent on the MSY gene sequence. The ddPCRs were carried out on C1000Touch (Bio-Rad) platform in 25 µl volume containing (final concentration) 1× ddPCR Supermix for Probes no-UTP, 10 µM forward and reverse primers for an MSY gene and the control gene, 250 nM TaqMan probe for an MSY gene and the control gene, one of the 2 restriction enzymes (diluted 1:1 in nuclease-free water), and 1–10 ng of undigested gDNA as a template. Droplets were generated using the QX200 (Bio-Rad) automated droplet generator and the manufacturer’s protocol. Cycling parameters were carried out using the recommended protocol for performing genomic enzymatic digestion during the PCR experiment. The PCR plate was transferred to QX200 (Bio-Rad) droplet reader and the data were analyzed using the associated QuantaSoft software. Analysis of ddPCR CNs per individual were determined by calculating the ratio of the male-specific target sequence concentration to the autosomal reference sequence concentration, times the number of copies of the reference sequence (CN = 2 for autosomal *MYOZI* and *MSTN*). The results were presented as either a whole integer or decimal number of copies per microliters of the final 1× ddPCR. The male control sample (Thoroughbred stallion *Bravo—*the DNA donor for the horse MSY assembly) (Janečka *et al.* 2018), the female control (Thoroughbred mare *Twilight—*the DNA donor for the horse reference genome) (Kalbfleisch *et al.* 2018), and a water control were present in all experiments (total 30 ddPCRs). All samples which CN results are reported in this study were tested a minimum in duplicates. Samples with questionable CN results (i.e. high SE, low droplet generation, or noticeably low or high CN) were retested until acceptable and consistent data were obtained (Supplementary Table 1). Samples not meeting these criteria were not included in the analysis. SE bars were generated by the QuantaSoft software using the Poisson distribution curve. Samples were considered to have a high SE when the Poisson confidence interval difference (Poisson maximum possible CN minus Poisson minimum CN) was greater than 1 copy. The number of droplets generated per well was calculated by the QuantaSoft software as the number of events. Samples with less than 8,000 total events were considered to have low droplet generation. As each horse was tested a minimum of 2 times for each gene, the final CNs presented in this study represent the mean for an individual or the mean per cohort. Gene CNs in all tables and supplementary tables are presented with decimals as in QuantaSoft output files. In the text, gene CNs are described and discussed as rounded values.

### CNV statistical analysis

Statistical analysis of gDNA CNVs between various cohorts was carried out using JMP v 15 (JMP 1989–2021). Because of sample size differences between breeds and cohorts, we combined parametric 1-way ANOVA with nonparametric Kruskal–Wallis test for generating *F*- and *H*-statistic *P-*values, respectively, to determine if there are statistically significant CNV within the 216-male cohort when divided by breed or Y-HT. Similar methods were used to compare the MSY gene CNs of 24 CO AQHs with those of the 28 AQHs in the normal cohort. Details of statistical analyses conducted and comparison between ANOVA and Kruskal–Wallis test results are presented in Supplementary Table 2. Due to heterogeneous phenotypes, *P-*values were not generated for the analysis of the 64, XY DSD cohort or the subfertile cohort. Instead, these individuals were compared with their corresponding breed group in the normal control cohort to identify outstanding CNVs potentially associated with the subfertility or DSD phenotype.

### MSY genotyping

We inferred MSY HTs of 216 male equids. For genotyping, we selected 30 HT determining variants as markers, from the previously described horse Y phylogeny (Felkel *et al.* 2019). Information about the variant markers—29 SNVs and 1 short indel, are given in Supplementary Table 4. The selected markers determine the major MS haplogroups (HGs) in the domestic horse population. Based on these markers, we created a condensed horse Y-HT tree, which served as a backbone for the HT analysis performed in this study.

gDNA was diluted with TE to a concentration of 5 ng/µl. For genotyping, competitive allele-specific PCR SNV genotyping assays (KASP, lgcgroup.com) were used. KASP genotyping was performed on a CFX96 Touch Real-Time PCR machine (BioRad) using the standard KASP genotyping protocol (LGC, Berlin, Germany). Each run included samples with their allelic state known as positive controls, while DNA from females and nontemplate controls were used as negative controls. Raw data were analyzed with Bio-Rad CFX Manager 3.1 software (BioRad).

Genotyping was conducted sequentially, following the hierarchical backbone tree. First, we determined whether samples belong to the Crown HG, which is the predominant HG in modern horse breeds, by genotyping the Crown determining variant rAX. If a sample carried the derived allele [C], which indicates that it carries an HT belonging to the Crown, clustering of the sample into Crown HGs T, A, and H was performed by testing variants rA, rW, and fYR. Based on the outcome, we genotyped the sample for the variants informative for the substructure of the HGs they cluster into. For samples carrying the ancestral allele at marker rAX [T], we genotyped 14 variants that determine the HGs outside the Crown in our backbone tree. For HT reconstruction, the information of the 30 markers were concatenated and allelic states of markers not tested, were imputed according to the HTs previously defined (Felkel *et al.* 2019) (see Supplementary Table 5). We constructed an HT frequency plot with draw.io platform (www.diagrams.net, 14.6.13; last accessed December 2021; https://github.com/jgraph/drawio). The phylogenetic relationships in the plot were based on the MSY tree from (Felkel *et al.* 2019), and the circle radiuses were scaled to the respective number of samples with RStudio 4.0.3 (RStudio 2020*)*.

## Results

### Horse MSY gene CN assays

We aimed to design ddPCR assays for all 15 multicopy genes, which are annotated in the current horse MSY assembly eMSYv3 (Janečka *et al.* 2018). However, in following assay requirements (Droplet Digital PCR Application Guide, BioRad) and MSY sequence properties, we were able to design assays for only 9 multicopy genes. Of these, the assays for 2 autosomal transposed genes—*HTRA3Y* and *SH3TC1Y*, were not male specific and hence, these genes were not used for CN analysis. In total, we succeeded to design and optimize male-specific ddPCR assays for 7 MSY multicopy genes. These included 4 amplified gametologs—*TSPY*, *RBMY*, *HSFY*, and *UBA1Y*, and 3 novel Y-born testis-specific transcripts—*ETSTY1*, *ETSTY2*, and *ETSTY5* (Janečka *et al.* 2018). In addition, ddPCR assay was successfully designed for the single-copy gene *SRY*. No copies of the 7 multicopy genes or the *SRY* gene were identified in the female control *Twilight*. Detailed information about the ddPCR assays used in this study is presented in Supplementary Table 3.

### Comparison of gene CNs inferred by ddPCR results and the MSY reference assembly eMSYv3

As the first step, we determined CNs of 7 multicopy genes and *SRY* in a multibreed cohort of 209 normal male horses and compared the results with the CNs in the horse MSY reference assembly, eMSYv3 (Janečka *et al.* 2018). The Thoroughbred *Bravo* was the DNA donor for eMSYv3 and the male control in all ddPCR experiments. Direct comparison between sequence-based and ddPCR-based CN evaluation was thus possible. For most genes, *Bravo’s* CNs determined by ddPCR were notably different from those in the MSY reference (Table 1). Five genes (*ETSTY2*, *ETSTY5*, *HSFY*, *TSPY*, and *UBA1Y*) had almost half as many copies by ddPCR compared with eMSYv3, while *ETSTY1* had 5 copies by ddPCR compared with 3 copies in eMSYv3. Only 2 genes, *SRY* (CN = 1) and *RBMY* (CN = 2) showed consistent rounded CN between ddPCR and the eMSYv3 reference.

**Table 1.** Comparison of gene CNs between eMSYv3 (Janečka *et al.* 2018) and ddPCR analysis of the reference male *Bravo* and a multibreed male horse cohort.

	*ETSTY1*	*ETSTY2*	*ETSTY5*	*HSFY*	*RBMY*	*SRY*	*TSPY*	*UBA1Y*
Male reference *Bravo* CN; eMSYv3 (Janečka *et al.* 2018)	3	7	8	3	2	1	13	8
Male reference *Bravo* mean CN*;* ddPCR	4.92; SD 0.26	4.58; SD 0.13	3.56; SD 0.05	1.09; SD 0.09	1.99; SD 0.03	0.90; SD 0.07	8.48; SD 0.24	3.48; SD 0.19
209 cohort mean CN	4.66	4.74	4.34	1.01	1.82	0.95	10.47	3.57
209 cohort minimum CN	1.96	2.8	1.88	0.22	0.6	0.46	5.55	1.12
209 cohort maximum CN	10.8	14	29	3	2.93	2.7	38	12.3
209 cohort SD	1.09	1.47	2.25	0.27	0.40	0.32	4.03	1.01

ddPCR data for the reference *Bravo* were generated from 30 technical replicates per gene and are presented with SD.

**Fig. 1.**
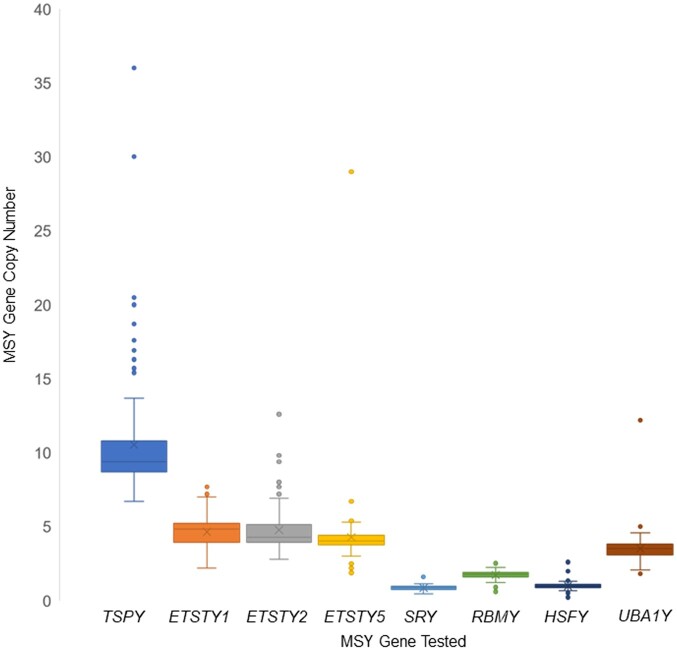
Box and whisker plot illustrating the range of CNV of horse MSY multicopy genes and *SRY* in a multibreed cohort of 209 normal male horses.

Similar disparities were observed when gene CNs in eMSYv3 reference were compared with mean gene CNs determined by ddPCR in a large multibreed cohort of 209 male horses, including *Bravo* (Table 1). At the same time, ddPCR-determined rounded mean gene CNs of *Bravo* and the 209-male cohort were identical for *ETSTY1*, *ETSTY5*, *HSFY*, *RBMY*, and *SRY* and very similar for *ETSTY2* (4 vs 5), *TSPY* (8 vs 10), and *UBA1Y* (3 vs 4). Due to an outlier (Yakutian; TR028; Supplementary Table 1) with exceptionally high CNs of multiple genes, the largest and smallest CNs per gene varied in a broad range, with *TSPY* having the largest range (from 5.5 to 38 copies) (Fig. 1, Table 1, and Supplementary Table 1). However, the outlier did not affect the overall mean CN in the population and was, therefore, not excluded from analysis. Regardless whether the outlier TR028 was included or not, *TSPY* remained the most variable multicopy gene tested in this study (Fig. 1).

### MSY gene CNV across horse breeds and related equids

The 209 normal male horse cohort comprised of 22 breeds or breed mixes allowing to compare MSY gene CNs across breeds (Table 2). Samples per breed ranged from a single individual (Friesian, American Paint, and Quarter Horse-Morgan mix) to 47 individuals for Thoroughbreds. The second most represented breeds were the Estonian Native horse and AQH with 28 individuals each. For each breed group, we calculated the mean CN along with SD and generated *F*-statistic (1-way ANOVA; *P1*) and *H*-statistic (Kruskal–Wallis; *P2*) *P-*values to determine if CNV between breeds was significant (Table 2). One-way ANOVA showed statistically significant (*P <*** **0.001) CN differences between breeds for multicopy genes *ETSTY1*, *ETSTY2*, *RBMY*, and *TSPY* and the single-copy *SRY*, though the studied horse breeds did not significantly differ for *ETSTY5*, *HSFY* and *UBA1Y* CNs (Table 2). The Kruskal–Wallis test, on the other hand, showed highly significant (*P <*** **0.001) CN variation of all genes across breeds, with a slightly lower significance (*P =*** **0.003) for *HSFY* (Table 2). The increase of statistical significance is likely attributed to the ability of this nonparametric test to control for the different number of individuals in different breeds. Though, both ANOVA and Kruskal–Wallis tests gave very similar results no matter whether breeds with less than 5 individuals were included or excluded (Supplementary Table 2). Regardless of the statistics used, the least CN variable genes across breeds were *HSFY* and *UBA1Y* and the most variable gene was *TSPY*, having a minimum of rounded 6 copies in the Friesian and a maximum of 17 copies in the Tennessee Walking horse (Table 2).

**Table 2.** Mean CN and corresponding SD of 7 MSY multicopy genes and *SRY* across horse breeds and related equids.

		*ETSTY1* ^a^	*ETSTY2* ^b^	*ETSTY5* ^b^	*HSFY*	*RBMY* ^b^	*SRY* ^b^	*TSPY* ^b^	*UBA1Y* ^a^
Horse breed	*N*	CN (SD)	CN (SD)	CN (SD)	CN (SD)	CN (SD)	CN (SD)	CN (SD)	CN (SD)
American Paint	1	3.60 (n/a)	4.48 (n/a)	4.26 (n/a)	1.04 (n/a)	1.77 (n/a)	0.90 (n/a)	9.80 (n/a)	3.48 (n/a)
Arabian	12	4.49 (0.77)	4.33 (1.09)	4.10 (0.37)	1.03 (0.17)	1.85 (0.20)	0.90 (0.09)	10.03 (3.43)	2.94 (0.87)
Caspian	6	4.73 (0.48)	4.53 (0.69)	4.08 (0.21)	0.97 (0.07)	1.88 (0.21)	0.84 (0.12)	9.67 (1.17)	3.57 (0.55)
Dales Pony	4	5.30 (1.58)	6.35 (0.79)	4.80 (0.51)	0.79 (0.22)	1.58 (0.49)	0.89 (0.16)	8.28 (0.59)	3.48 (0.40)
Estonian Native	28	4.49 (1.26)	5.93 (2.48)	5.76 (5.90)	1.15 (0.48)	2.15 (0.43)	1.21 (0.46)	12.98 (7.04)	4.09 (2.39)
Friesian	1	1.96 (n/a)	3.78 (n/a)	2.62 (n/a)	0.85 (n/a)	1.91 (n/a)	0.78 (n/a)	5.55 (n/a)	3.13 (n/a)
Haflinger	2	4.12 (0.13)	3.73 (0.06)	4.07 (0.02)	0.99 (0.02)	1.99 (0.16)	0.96 (0.07)	9.25 (0.35)	4.11 (0.23)
Icelandic	6	4.83 (0.26)	3.94 (0.47)	4.05 (0.23)	1.01 (0.08)	1.77 (0.25)	0.90 (0.15)	9.48 (0.86)	3.71 (0.37)
Lipizzan	10	5.10 (0.36)	3.85 (0.28)	3.90 (0.09)	0.95 (0.09)	1.91 (0.17)	0.96 (0.03)	8.73 (0.31)	3.99 (0.22)
Miniature	2	3.71 (0.08)	3.91 (0.01)	3.94 (0.34)	0.82 (0.01)	1.63 (0.17)	0.86 (0.08)	8.95 (0.49)	3.45 (0.05)
Mongolian	10	4.46 (0.54)	4.16 (0.30)	3.79 (0.23)	0.96 (0.11)	1.67 (0.55)	1.26 (0.51)	8.47 (0.84)	3.74 (0.34)
Noriker	4	4.91 (0.65)	3.92 (0.12)	3.89 (0.05)	0.98 (0.07)	1.92 (0.11)	0.98 (0.06)	8.68 (0.49)	4.00 (0.08)
Quarter Horse	28	4.64 (0.75)	4.59 (0.85)	4.21 (0.49)	1.00 (0.15)	1.79 (0.23)	0.83 (0.11)	10.66 (2.62)	3.43 (0.52)
Quarter Horse-Morgan mix	1	3.59 (n/a)	4.50 (n/a)	4.12 (n/a)	0.98 (n/a)	1.79 (n/a)	0.93 (n/a)	10.00 (n/a)	3.51 (n/a)
Shetland pony	5	5.10 (0.20)	4.25 (0.69)	4.21 (0.44)	0.94 (0.05)	1.73 (0.43)	0.80 (0.27)	8.37 (0.50)	3.80 (n/a)
Standardbred	8	5.08 (1.04)	5.11 (1.00)	4.62 (0.94)	1.11 (0.17)	1.41 (0.27)	0.61 (0.08)	9.00 (1.16)	2.98 (0.24)
Suffolk Punch	8	3.89 (0.17)	4.50 (0.31)	4.12 (0.24)	1.02 (0.11)	1.87 (0.13)	0.82 (0.09)	9.32 (1.19)	3.44 (0.32)
Heck horse (Heck 1952)	7	5.86 (1.26)	6.17 (0.81)	4.99 (0.40)	1.10 (0.08)	1.34 (0.46)	0.74 (0.11)	15.36 (2.64)	3.56 (0.45)
Tennessee Walking	5	5.60 (1.11)	6.02 (1.04)	4.61 (0.36)	1.07 (0.15)	1.62 (0.18)	0.79 (0.22)	16.92 (2.59)	3.56 (0.64)
Thoroughbred	47	4.49 (0.91)	4.35 (0.75)	3.74 (0.62)	0.91 (0.27)	1.79 (0.21)	0.87 (0.14)	9.35 (1.19)	3.50 (0.63)
Yakutian	4	5.97 (3.37)	6.41 (5.07)	5.58 (3.42)	1.22 (0.65)	1.78 (0.31)	1.40 (0.87)	15.72 (14.88)	3.59 (0.52)
Zemaitukai	10	5.39 (0.41)	5.02 (0.76)	4.70 (0.32)	0.92 (0.08)	2.32 (0.54)	1.27 (0.42)	11.62 (1.09)	3.62 (0.28)
*P1-*value; *F*-statistic	209	0.0003	<0.0001	0.64	0.19	<0.0001	<0.0001	<0.0001	0.59
*P2*-value; *H*-statistic	209	0.0002	<0.0001	<0.0001	0.003	<0.0001	<0.0001	<0.0001	0.001
Difference between Max and Min CN		4.01	2.68	3.14	0.43	0.98	0.79	11.37	1.17

**Equid species**	***N***	***ETSTY1***	***ETSTY2***	***ETSTY5***	***HSFY***	***RBMY***	***SRY***	***TSPY***	***UBA1Y***

Przewalski's horse	5	3.65 (1.85)	3.18 (0.40)	4.11 (0.31)	1.02 (0.15)	1.10 (0.23)	0.93 (0.14)	8.40 (0.77)	3.04 (0.58)
Kulan	2	2.29 (0.25)	4.10 (0.85)	3.50 (0.85)	1.69 (0.03)	0.82 (0.09)	0.75 (0.13)	n/a	n/a
Horse mean from Table 1	209	4.66	4.74	4.34	1.01	1.82	0.95	10.47	3.57

CNs of Przewalski’s horse and kulan are presented separately as an outgroup.

*N*, number of individuals.

a *P *<* *0.001.

b *P *<* *0.0001.

Interestingly, *SRY* was a single-copy gene in most breeds and individuals used in this study (Table 2 and Supplementary Table 1), though we identified 21 individuals from 4 indigenous breeds (Estonian Native horse, Mongolian, Yakutian, and Zemaitukai) with 2 or 3 copies of *SRY* (Table 3 and Supplementary Table 1). Most of these 21 individuals had also an increased number of *RBMY* copies (CN = 3), resulting in significant (*P <*** **0.0001) *SRY* and *RBMY* CN differences between breed groups (Table 2). However, 3 of the 21 males had a decreased *RBMY* CN = 1 (Table 3).

**Table 3.** Individuals from 4 indigenous breeds with deviation in CNV of *SRY* (expected CN = 1) and *RBMY* (expected CN = 2).

Breed	Horse ID	*SRY* CN	*RBMY* CN
Estonian Native	BP364	1.96	2.81
Estonian Native	BP378^a^	2.0	3.0
Estonian Native	BP379	1.74	2.685
Estonian Native	BP383	1.88	2.92
Estonian Native	BP384	1.49	2.66
Estonian Native	BP385	1.9	2.5
Estonian Native	BP386^a^	1.58	2.35
Estonian Native	BP387^a^	1.77	2.6
Estonian Native	BP388^a^	1.55	2.85
Estonian Native	BP395^a^	1.6	2.52
Estonian Native	BP399^a^	1.76	2.53
Estonian Native	BP400	1.98	2.61
Mongolian	BP298	1.99	2.785
Mongolian	TR020	1.87	1.06
Mongolian	TR021	1.95	1.01
Yakutian	TR028	2.7	1.37
Zemaitukai	121576^a^	1.7	2.8
Zemaitukai	121579	1.48	2.84
Zemaitukai	121581	1.66	2.77
Zemaitukai	121587	1.58	2.79
Zemaitukai	121589	1.89	2.93

a Confirmed fertile breeding stallions.

In addition to the breeds of the domestic horse (*Equus caballus*), we used the optimized ddPCR assays for MSY gene CN analysis in 2 other equid species—Przewalski’s horse (*Equus przewalskii*)—a caballine closely related to the domestic horse, and the kulan (*Equus hemionus kulan*)—an equid from the ass/onager group (Table 2). Assays for all 8 genes worked in Przewalski’s horse and showed CNs similar to or lower than domestic horse mean values (Table 2). In the kulan, ddPCR results were obtained for 6 genes, while the assays for equine *TSPY* and *UBA1Y* did not work (Table 2), likely due to MSY sequence divergence. Both wild equids, like most domestic horses, had a single copy of the *SRY* gene.

### MSY HT analysis

**Fig. 2.**
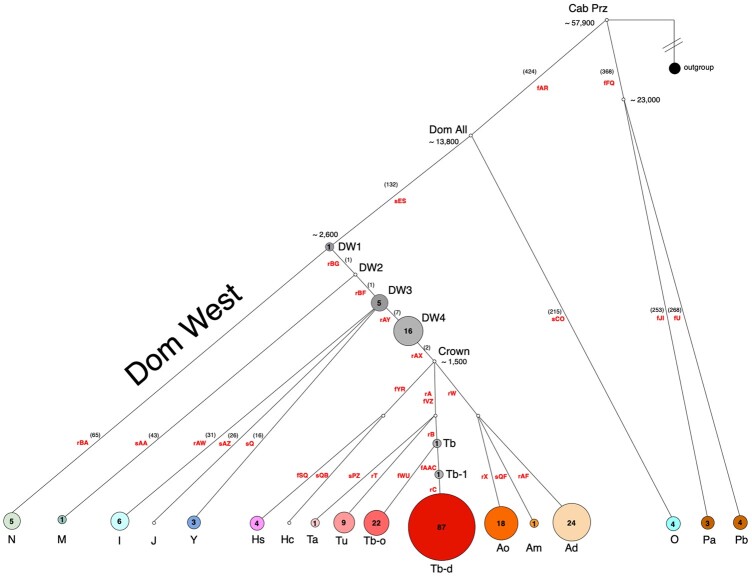
HT distribution and frequency plot based on MSY tree modified from (Felkel *et al.* 2019). HTs are given as circles with HT symbols below and circle radius proportional to the number of clustered individuals. Absolute number of individuals are given inside the circles. Different colors and shades correspond to HTs represented in the dataset, while noncolored points express HTs that were not detected in the sample set. Number of mutations on non-Crown branches in Felkel *et al.* (2019) are denoted in brackets; in Crown HG they ranged from 5 to 26. Markers used for genotyping are in red font; Domestic West is abbreviated as DW. Estimated splitting times for branching points (years before present) are from Felkel *et al.* (2019).

We genotyped 30 MSY polymorphic SNV markers (Supplementary Table 4) in 209 normal male horses, 5 Przewalski’s horses, and 2 kulans (outgroup) and assigned individuals to HGs and HTs according to Felkel *et al.* (2019). The 209 domestic horses and 5 Przewalski’s horses separated into 20 HTs (Fig. 2 and Supplementary Table 5). Consistent with expectations, the kulans formed an outgroup. We assigned 190 horses (including Przewalski’s horses) to 14 previously defined HTs and 24 males were placed into internal nodes of the backbone topology Domestic West 1 (DW1), DW2, DW3, DW4, Tb, and Tb-1 (Fig. 2). The 24 inner clustering samples carry not yet resolved HTs, branching off at the respective node with their private SNVs unknown.

Most domestic horses (79%; 166/209) clustered into the Crown HG (Fig. 2 and Supplementary Table 5). Within the Crown HG samples, we distinguished 10 HTs (including 2 not fully characterized HTs Tb and Tb-1). The most represented HT was Tb-d (*n* = 87) which encloses 45 of 46 analyzed Thoroughbreds, but also 23 Quarter Horses, 8 Standardbreds, 5 Tennessee Walking horses, 3 Caspian ponies, 1 Paint, 1 Quarter-Morgan mix, and 1 Estonian Native horse. The next abundant HTs were Ad and Tb-o with 24 and 21 horses, respectively. Crown HT Tu comprised of 8 Estonian Native horses and Hs made up of 4 Lipizzans. A single Caspian horse had Am and a single Zemaitukai horse had Ta HTs. Details about individuals, breeds and corresponding HGs and HTs are presented in Supplementary Table 5.

Out of 209 genotyped horses, 43 (21%) carried the ancestral allele for the rAX variant (Felkel *et al.* 2019), and were placed outside the large Crown HG and categorized as “non-Crown.” Nordic breeds belonged to I and N HGs, and Asian horses grouped into O, M, and Y HGs. Six Mongolian horses could not be attributed to any ascertained HTs and were clustered basally into DW1 (*n* = 1) and DW3 (*n* = 5). Finally, 11 Estonian Native and 5 Zemaitukai horses clustered into DW4 having a derived allele at rAY and the ancestral allele at the rAX variant (Fig. 2).

As expected, the 5 Przewalski’s horses separated from domestic horses and fell into the previously identified P HG with Pa and Pb HTs (Felkel *et al.* 2019) (Fig. 2). Interestingly, while the Pb HT was noted in 4 Przewalski’s horses, the Pa HT was carried by a single Przewalski’s horse and 2 Heck horses which were developed through the integration of Przewalski’s horses into a domestic horse (Heck 1952; Lovász *et al.* 2021; Supplementary Table 5).

### MSY gene CNV across MSY HTs

For each of the 21 MSY HTs (including the kulan outgroup) we generated a mean CN of MSY genes (Table 4) and used both *F*- and *H*-statistics to determine if there was a significant CNV between HTs. Similarly to gene CNV across breeds (Table 2), highly significant (*P <*** **0.001) CN differences between HTs were observed for most genes, except *ETSTY5, HSFY*, and *UBA1Y* (Table 4). Like in the breed comparison, *TSPY* had the broadest range of variation with a rounded minimum CN = 8 in the Y HT and rounded maximum CN = 17 in the Tu HT. Interestingly, a mean rounded CN of *SRY* in O HT was 2 and CN of *RBMY* in DW4 was 3. However, while MSY gene CN was significantly different between MSY HTs, we did not observe any correlation between the patterns of the 2 types of variation.

**Table 4.** Mean CN and SD of 7 multicopy genes and *SRY* in 20 MSY HTs and the kulan outgroup.

MSY HG	MSY HT	No of horses	*ETSTY1* ^b^	*ETSTY2* ^b^	*ETSTY5*	*HSFY*	*RBMY* ^b^	*SRY* ^b^	*TSPY* ^a^	*UBA1Y*
			CN (SD)	CN (SD)	CN (SD)	CN (SD)	CN (SD)	CN (SD)	CN (SD)	CN (SD)
Crown	Ad	24	4.06 (1.15)	4.88 (1.03)	4.98 (3.85)	1.02 (0.37)	1.88 (0.32)	0.89 (0.17)	10.07 (4.05)	3.87 (1.83)
Crown	Am	1	4.15 (n/a)	5.49 (n/a)	4.23 (n/a)	0.98 (n/a)	1.77 (n/a)	0.79 (n/a)	8.60 (n/a)	3.21 (n/a)
Crown	Ao	18	4.75 (0.73)	4.21 (0.9)	4.05 (0.31)	0.99 (0.16)	1.88 (0.18)	0.93 (0.09)	9.60 (2.83)	3.29 (0.87)
Crown	Ta	1	5.40 (n/a)	5.20 (n/a)	4.90 (n/a)	0.95 (n/a)	1.63 (n/a)	0.89 (n/a)	11.80 (n/a)	3.70 (n/a)
Crown	Tb	1	5.14 (n/a)	3.90 (n/a)	4.35 (n/a)	0.91 (n/a)	1.67 (n/a)	0.65 (n/a)	10.50 (n/a)	4.00 (n/a)
Crown	Tb-1	1	4.14 (n/a)	4.60 (n/a)	3.80 (n/a)	0.92 (n/a)	1.81 (n/a)	0.82 (n/a)	10.30 (n/a)	2.67 (n/a)
Crown	Tb-d	87	4.64 (0.93)	4.59 (0.89)	4.02 (0.67)	0.96 (0.23)	1.75 (0.25)	0.83 (0.15)	10.09 (2.51)	3.43 (0.59)
Crown	Tb-o	21	4.89 (0.59)	4.85 (1.22)	4.28 (0.50)	1.01 (0.13)	1.81 (0.23)	0.89 (0.20)	11.15 (2.39)	3.61 (0.37)
Crown	Tu	8	4.75 (1.57)	8.41 (3.26)	7.60 (8.67)	1.34 (0.63)	1.90 (0.37)	0.96 (0.32)	16.79 (10.92)	4.73 (3.09)
Crown	Hs	4	4.87 (0.46)	3.86 (0.43)	3.84 (0.05)	0.98 (0.06)	1.85 (0.22)	0.96 (0.03)	8.59 (0.44)	3.93 (0.35)
Non-Crown	DW1	1	4.90 (n/a)	4.40 (n/a)	3.90 (n/a)	0.87 (n/a)	1.83 (n/a)	0.92 (n/a)	8.44 (n/a)	3.40 (n/a)
Non-Crown	DW3	5	4.04 (0.43)	4.28 (0.28)	3.65 (0.15)	0.95 (0.04)	1.96 (0.47)	1.12 (0.49)	8.39 (1.2)	3.63 (0.36)
Non-Crown	DW4	16	4.98 (0.95)	4.82 (0.76)	4.20 (0.59)	0.99 (0.17)	2.58 (0.36)	1.62 (0.34)	11.25 (2.16)	3.49 (0.31)
Non-Crown	Y	3	4.35 (1.22)	3.87 (0.26)	3.87 (0.29)	0.90 (0.02)	1.91 (0.19)	0.97 (0.11)	8.29 (1.13)	3.55 (0.63)
Non-Crown	I	6	4.83 (0.26)	3.94 (0.47)	4.05 (0.23)	1.01 (0.08)	1.77 (0.25)	0.90 (0.15)	9.48 (0.86)	3.71 (0.37)
Non-Crown	M	1	4.76 (n/a)	3.87 (n/a)	3.89 (n/a)	0.99 (n/a)	2.02 (n/a)	0.98 (n/a)	8.40 (n/a)	4.14 (n/a)
Non-Crown	N	5	5.10 (0.20)	4.25 (0.69)	4.21 (0.44)	0.94 (0.05)	1.73 (0.43)	0.80 (0.27)	8.37 (0.50)	3.80 (0.30)
Non-Crown	O	4	6.38 (2.95)	6.48 (5.02)	5.63 (3.39)	1.31 (0.62)	1.11 (0.18)	2.17 (0.46)	15.97 (14.69)	3.85 (0.21)
Non-Crown	Pa	3	7.32 (0.38)	5.87 (1.81)	4.91 (0.76)	1.05 (0.16)	0.96 (0.47)	0.80 (0.16)	15.07 (5.53)	3.63 (0.24)
Non-Crown	Pb	4	2.83 (0.12)	3.03 (0.23)	4.12 (0.35)	1.04 (0.17)	1.00 (0.06)	0.92 (0.15)	8.33 (0.87)	2.83 (0.40)
Non-Crown	Outgroup	2	2.29 (0.25)	4.10 (0.85)	3.50 (0.85)	1.69 (0.03)	0.82 (0.09)	0.75 (0.13)	n/a	n/a
*P1*-value; *F*-statistic		203	<0.0001	<0.0001	0.2436	0.0183	<0.0001	<0.0001	0.0014	0.366
*P2*-value; *H*-statistic		203	0.0004	0.0001	0.085	0.56	<0.0001	<0.0001	0.001	0.213

a *P *<* *0.001.

b *P *<* *0.0001

### MSY gene CNV in cryptorchid males, XY horses with various forms of DSD and in subfertile/infertile males

We investigated MSY gene CNs in 3 groups of abnormal male horses. The first group consisted of 24 AQHs with bi-CO (*n* = 6) or uni-CO (*n* = 18). MSY CNs were compared within this group (uni-CO vs bi-CO) as well as with the 28 normal AQHs from the large male cohort (Table 2). Here, both *F*- and *H*-statistics produced highly concordant results. There was no significant CN difference between bi-CO and uni-CO, nor between bi-CO and the normal cohort (Table 5). However, there was a significant (*P <*** **0.05) CN difference in *TSPY*, *SRY*, and *RBMY* between uni-CO and normal males. CNs of the same 3 genes and *ETSTY2* were significantly different between all CO and normal AQHs (Table 5). Though, compared with the statistical significance of gene CNs across breeds (*P <*** **0.001) and HTs (*P <*** **0.001), the significance level of differences between CO and normal AQHs was a magnitude lower (*P <*** **0.05). The *TSPY* gene showed the most notable CN change having 2 copies less in CO (rounded mean CN = 9) than normal males (rounded average CN = 11). The rounded mean CN of *ETSTY2* was 4 in CO compared with 5 copies in normal males. It must be noted that while statistical analysis showed significant CN differences also for *SRY* and *RBMY*, their rounded mean CNs in CO and normal males were the same—1 copy for *SRY* and 2 copies for *RBMY* (Table 5). The reason while statistics gives significant difference to the same rounded CN is that ddPCR is a continuous measurement and gives noninteger values for a biologically discrete measure—the gene CN with integer values. When statistical analysis is done for low CN genes, like *SRY* or *RBMY*, any subtle difference in decimals can create statistical significance when comparing the means between populations. Thus, it is not the fault in statistics but rather the nature of rounding CN for low CN genes. Individual CNs for the 52 AQHs used for this analysis are presented in Supplementary Tables 1 and 6.

**Table 5.** Comparison of MSY gene mean CN between cryptorchid and normal AQHs.

Horse groups	*N*	*ETSTY1*	*ETSTY2* ^a^	*ETSTY5*	*HSFY*	*RBMY* ^a^	*SRY* ^b^	*TSPY* ^a^	*UBA1Y*
Bi-CO	6	4.16	4.22	4.01	1.03	1.92	0.9	9.11	3.63
Uni-CO	18	4.69	4.17	4.21	1.06	1.95	0.96	9.22	3.7
CO all	24	4.55	4.18	4.16	1.05	1.94	0.94	9.19	3.68
Normal AQHs	28	4.67	4.58	4.21	0.99	1.78	0.83	10.65	3.42
*P1-*value; *F*-statistic									
Bi-CO vs Uni-CO		0.4708	0.7998	0.5978	0.6795	0.85497	0.4017	0.883	0.7249
Bi-CO vs normal		0.1338	0.3067	0.3576	0.6445	0.1896	0.1484	0.167	0.3688
Uni-CO vs normal		0.9654	0.0642	0.9794	0.2206	**0.0467**	**0.0027**	**0.045**	0.0746
CO all vs normal		0.7213	**0.0392**	0.7595	0.2208	**0.0303**	**0.0025**	**0.019**	0.0583
*P2*-value; *H*-statistic									
Bi-CO vs Uni-CO		0.641	0.351	0.894	0.368	0.92	0.424	0.571	0.689
Bi-CO vs normal		0.136	0.32	0.249	0.668	0.175	0.136	0.183	0.498
Uni-CO vs normal		0.208	0.057	0.26	0.15	**0.044**	**0.004**	**0.029**	0.129
CO all vs normal		0.102	**0.051**	0.166	0.177	**0.028**	**0.003**	**0.02**	0.13

*P*-values in bold are statistically significant.

a *P *<* *0.05.

b *P *<* *0.005.

The second abnormal group of horses comprised of 29 individuals from 7 breeds or breed mixes with 64, XY karyotype and various forms of DSDs (Supplementary Table 7). Of these, 4 individuals had cytogenetically detectable Y chromosomal deletions (64, XYdel) and female-like or intersex phenotypes, twelve individuals were XY females with *SRY*-negative male-to-female sex reversal condition (Raudsepp *et al.* 2010; Bugno-Poniewierska and Raudsepp 2021), 6 were XY female-like horses with *SRY*-positive sex reversal, and 7 individuals were phenotypically intersex with normal *SRY*-positive male karyotype.

None of the 8 ddPCR assays amplified in the 4 individuals with cytogenetically detectable Y deletions, indicating a complete loss of these sequences. All twelve XY *SRY*-negative sex reversal females, in addition to the missing *SRY*, had only 1 copy of *RBMY* instead of the expected 2 copies, suggesting that 1 copy of *RBMY* was lost together with *SRY*. The remaining 13 horses with *SRY*-positive XY DSDs did not show noticeably higher or lower CNs for the genes tested when compared with their corresponding breed group mean in the large male cohort (Table 2). Individual CNs for the DSD group are presented in Supplementary Tables 1 and 7.

The third group of abnormal horses comprised of 14 male horses of 6 breeds with variable subfertility/infertility phenotypes (Supplementary Table 8). Comparison of individual MSY CNs in this group to their breed mean value in the large normal male cohort (Table 2) did not reveal any significant differences. However, we noticed that CNs of 2 Arabians in this group slightly deviated from breed mean: 1 (H963) with idiopathic subfertility and autosomal translocation had more copies of *TSPY* (rounded CN = 14 vs 10), *ETSTY2* (rounded CN = 5 vs 4), and *ETSTY5* (rounded CN = 6 vs 4), while another (H284) with idiopathic subfertility, had less copies of *UBA1Y* (rounded CN = 1 vs 3). Individual CNs for the subfertile/infertile male group are presented in Supplementary Tables 1 and 8.

### MSY gene CNs and HTs of closely related males

Within the 209 normal male cohort, we identified 4 sets of directly related male individuals with available MSY CN and HT information. These included 2 sire–son pairs, 1 grandsire–son pair, and 1 grandsire–sire–son trio. In addition, MSY CN data were generated for 2 cloned Arabians (group 5) and 6 cloned AQHs (group 6), all produced by SCNT (Table 6). This allowed us to investigate the dynamics of MSY gene CN and HT between generations and MSY CN across genetically identical individuals.

**Table 6.** MSY gene CN and HT comparison between related males.

Group	Relation	Breed	HG	HT	*ETSTY1*	*ETSTY2*	*ETSTY5*	*HSFY*	*RBMY*	*SRY*	*TSPY*	*UBA1Y*	Horse ID
1	Grand-sire	Estonian Native	Non-Crown	DW4	5.3	6	3.7	0.94	2.6	1.77	15.8	3.5	BP387
1	Sire	Estonian Native	Non-Crown	DW4	6.8	4.9	4	0.79	3	2	14.7	3.18	BP378
1	Son	Estonian Native	Non-Crown	DW4	3.8	3.7	n/a	n/a	2.81	1.96	10.1	n/a	BP364
2	Grand-sire	Estonian Native	Crown	Ad	4	5.4	4.6	0.88	1.92	0.79	11.7	2.9	BP282
2	Son	Estonian Native	Crown	Ad	5.6	4.5	4	0.99	2.85	1.55	12.9	3.07	BP388
3	Sire	Estonian Native	Non-Crown	DW4	5	5	3.3	1.23	2.53	1.76	10.4	3.06	BP399
3	Son	Estonian Native	Non-Crown	DW4	4.4	4.2	3.7	0.94	2.61	1.98	9.2	3.7	BP400
4	Sire	Heck horse	Non-Crown	Pa	7.3	7.2	5.4	1.17	0.6	0.68	17.8	3.6	15758
4	Son	Heck horse	Non-Crown	Pa	7.7	6.6	5.3	1.05	0.79	0.73	18.7	3.4	21150
5	Cloned brother	Arabian	n/a	n/a	4.37	4.62	4.73	1.07	1.74	0.99	11.9	3.11	H962
5	Cloned brother	Arabian	n/a	n/a	4.27	4.87	5.86	0.99	1.7	0.86	14.2	3.1	H963
6	SCNT Donor	AQH	n/a	n/a	4.9	5.6	4.52	0.93	1.89	0.77	14.3	3.2	H396
6	Cloned brother	AQH	n/a	n/a	4.69	4.6	4.41	1.06	1.69	0.84	12.8	3.35	H391
6	Cloned brother	AQH	n/a	n/a	4.55	5.2	4.09	0.98	1.68	0.79	12	3.01	H392
6	Cloned brother	AQH	n/a	n/a	4.54	5.3	4.32	1.02	1.54	0.77	14.4	2.98	H393
6	Cloned brother	AQH	n/a	n/a	4.29	4.9	4.45	1.15	1.79	0.8.	13.5	3.1	H394
6	Cloned brother	AQH	n/a	n/a	4.44	5.1	4.44	0.96	1.92	0.78	11.6	2.85	H395

According to expectation, MSY HTs were conserved in all patrilines (Table 6). In contrast, MSY gene CNs were not conserved, and we observed duplications and/or deletions in every generation including the cloned horses.

In group 1 (Table 6), we determined that *TSPY* underwent major deletions between the 3 generations, losing 6 copies from grand sire (CN = 16) to son (CN = 10). Likewise, *ETSTY2* lost 2 copies over 3 generations—from rounded CN = 6 in grand sire, CN = 5 in sire to CN = 4 in son. Different dynamics were observed for *ETSTY1*, which had a CN increase from grand sire (5 copies) to sire (7 copies) but reduced to 4 copies in son. CNs of *SRY* (CN = 2) and *RBMY* (CN = 3), however, remained the same over the 3 generations. Because of limited amount of DNA, we were not able to obtain CN data for *ETSTY5*, *HSFY*, and *UBA1Y* for the son (BP364), though CNs of these 3 genes did not differ between the grand sire and sire.

In group 2 (Table 6), we observed a CN increase of 2 in *ETSTY1* from grand sire to son, and a CN increase of 1 in *TSPY*, *SRY*, and *RBMY*, and a 1 copy decreases in *ETSTY2* and *ETSTY5*. No CN changes were observed for *HSFY* and *UBA1Y*. Here, the most intriguing transgenerational CN change was for *SRY* and *RBMY* because most male horses have 1 copy of *SRY* and 2 copies of *RBMY*, as confirmed by ddPCR (Table 1) and presented in the MSY reference assembly (Janečka *et al.* 2018).

Groups 3 and 4 were both sire–son pairs, and we observed more MSY CNV in group 3 over a single generation (Table 6): *TSPY*, *ETSTY1*, and *ETSTY2* each lost 1 copy and *ETSTY5* and *UBA1Y* each gained 1 copy from the sire to son. MSY CNs were more stable in group 4, where the only difference between generations was an extra copy of *TSPY* in the son.

Group 5 comprised of genetically identical Arabians derived from the same somatic cell donor (DNA not available) by SCNT. The 2 clones differed by 1 copy for *ETSTY5* and 2 copies for *TSPY*. In group 6, we compared CN of 5 cloned AQHs and their somatic cell donor and observed a range of CNV for *TSPY* (12–14 copies) and some CN differences for *ETSTY1* and *ETSTY2* (Table 6).

## Discussion

Here, we present the first comprehensive ddPCR-based CNV analysis of 7 horse MSY multicopy genes and *SRY*. We established a baseline CN for these genes, allowing critical evaluation of the current horse MSY sequence assembly eMSYv3 (Janečka *et al.* 2018) and to study MSY gene CNV in large horse populations, and males with DSD and reproduction. For the first time, the dynamics of horse MSY variation was compared at gene CNV and SNV levels.

CN analysis of genes in the structurally complex Y chromosome relies heavily on the availability of a high-quality reference assembly. The first annotated reference sequence of the horse MSY, eMSYv3 (Janečka *et al.* 2018), presents a high-quality assembly of single-copy regions but remains tentative for the ampliconic MSY—the region where multicopy genes reside. The tentative nature of the ampliconic MSY assembly complicated the design of ddPCR assays for CNV analysis in this and previous studies (Castaneda *et al.* 2019, 2021) where one of the main limitations was inability to find a single shared male-specific sequence across all copies of a gene. Therefore, we were able to develop CN assays for only 7 (*ETSTY1*, *ETSTY2*, *ETSTY5*, *HSFY, RBMY*, *TSPY*, and *UBA1Y*) out of the 15 known horse MSY multicopy genes (Janečka *et al.* 2018), as well as for the single-copy *SRY*. The study of the remaining 8 multicopy genes will require substantial improvement of the assembly of the ampliconic region of horse MSY.

In a way, improvement of the current eMSYv3 reference already started in this study by comparing CNs of 8 MSY genes in the same individual horse—a Thoroughbred stallion *Bravo* who was the DNA donor for eMSYv3 (Janečka *et al.* 2018) and the reference male for all ddPCR experiments (Table 1). Since multiple ddPCR experiments gave consistent CNs for MSY genes and autosomal control genes in the reference horse (Supplementary Table 1), we considered ddPCR results reliable. It is therefore noteworthy that only 2 genes, *SRY* and *RBMY*, both located in a transitional region between single-copy and multicopy MSY (Janečka *et al.* 2018), had the same CN in eMSYv3 and by ddPCR (Table 1) confirming correct assembly of this MSY region. In contrast, *ETSTY2*, *ETSTY5*, *HSFY*, *TSPY*, and *UBA1Y* had almost twice as many copies in eMSYv3 than detected by ddPCR, suggesting over-assembly of the corresponding regions. Conversely, slightly lower CN for *ETSTY1* in eMSYv3 (CN = 3) compared with ddPCR (CN = 5) indicated that the MSY reference is likely missing some copies of this equine testis-specific transcript. While our findings strongly support the accuracy of ddPCR results over eMSYv3, we cannot exclude that the designed ddPCR assays did not target all copies of some genes due to incomplete or diverged sequences. In addition, as observed for CN analysis of *SRY* and *RBMY* between normal and cryptorchid AQHs (Table 5 and Supplementary Table 6), statistical analysis of very similar noninteger CN values of low CN genes may produce true statistical significance between study cohorts, even though the rounded integer CNs are the same in all individuals. This is an inherent detriment of using ddPCR-based continuous measurement for measuring gene CN which is a discrete value. While we acknowledge this limitation, the solution would be to increase sample size for each cohort, which was beyond the scope and sample availability in this study.

The development of CN assays and determining baseline CN for a few MSY genes in the reference male *Bravo*, allowed to expand CN analysis to large multibreed horse populations and related equids. To date, this is the most extensive MSY gene CN study in equids encompassing 282 domestic horses (209 normal and 73 with disorders) from 22 breeds, Przewalski’s horse and kulan (Supplementary Table 1). The only study of similar scope has been conducted in 263 donkeys of 13 breeds (Han *et al.* 2017) where CNs of 5 MSY genes (*CUL4BY*, *ETSTY1*, *ETSTY4*, *ETSTY5*, and *SRY*) were evaluated by qPCR which is a relative quantitation method. Due to different methodological approaches (qPCR vs ddPCR), the results of the donkey study are too different for any meaningful comparison with our data. For example, the donkey study documented *SRY* CN range from 1 to 152. This is in stark contrast with this study where we show that *SRY*, together with *RBMY*, were the only genes with consistent mean CN across all study cohorts and the eMSYv3 reference (Tables 1 and 2). Additional support for the accuracy of ddPCR was the fact that mean CNs of the remaining 6 genes were the same (*ETSTY1, ETSTY5, HSFY*) or similar (*ETSTY2, TSPY, UBA1Y*) between the reference male *Bravo* and the multibreed cohort of 209 normal horses (Table 1). Also, previous studies have indicated high degree of cytogenetic and sequence conservation between the horse and donkey MSYs (Paria *et al.* 2011). Therefore, it is unlikely that the CN differences between this study and that by Han *et al.* (2017) were caused by extensive divergence of equine and asine Y chromosomes. This is further supported by our results in the kulan, another equid from the asine group, where *SRY* CN was consistently 1 (Table 2).

Much more gene CNV was observed when the 209-horse cohort was broken down into breeds showing statistically significant CN differences for 5 of the 8 genes studied by *F*-statistics and for all genes by *H*-statistics (Table 2 and Supplementary Table 2). The latter (Kruskal–Wallis test) was used to help to account for the differences in sample sizes representing different horse breeds (from 1 Friesian to 47 Thoroughbreds; Table 2), though both statistical approaches gave overall highly concordant results and were not influenced by the inclusion or exclusion of breeds with *n* < 5 individuals (Tables 2, 4, and 5 and Supplementary Table 2). Notably, significant interbreed CN differences of *SRY* and *RBMY* were exclusively caused by a few individuals from indigenous breeds (Table 3). Otherwise, CNs of these 2 genes were stable across most breeds (Table 1) and individuals (Supplementary Table 1). Likewise, significant interbreed CNV of *ETSTY2* was caused by 1 Yakutian (TR028) and 2 Estonian Native horses (BP379 and BP380), having 14 or 13 copies (Supplementary Table 1), respectively, compared with the horse cohort mean of 5 (Table 2). In fact, the same Yakutian horse (TR028) showed extremely high CNs for all genes studied, except *RBMY*, suggesting that the horse may have a cytogenetic abnormality with an extra Y chromosome. Though, we could not verify this because cytogenetic information was available only for the 73 abnormal males (Supplementary Table 1) but not for most individuals in the 209-horse cohort. Regardless of the inclusion of sample TR028, the truly most CN variable gene across breeds and individuals was *TSPY*, the gene which also had the highest CN (mean 10, lowest 6, highest 38) among all MSY genes (Tables 1 and 2 and Supplementary Table 1). Higher variability between individuals within larger ampliconic gene families (specifically *TSPY*) have also been reported in humans (Skov et al. 2017; Lucotte *et al.* 2018; Ye *et al.* 2018; Vegesna *et al.* 2019) and great apes (Oetjens *et al.* 2016; Tomaszkiewicz *et al.* 2016; Vegesna *et al.* 2020) and is because multicopy genes with higher CN have an increased probability of being involved in intrachromosomal rearrangements compared with genes with lower CNs (Ghenu *et al.* 2016). In this context, *TSPY* is also a good example for other species because it is a multicopy gene in nearly all mammalian Y chromosomes (Bellot *et al.* 2014; Cortez *et al.* 2014), but shows different degree of CNV in different species depending on the baseline CN. For example, the estimated CN of cattle *TSPY* is 50–200 and the gene shows significant CNV between individuals, breeds, and subspecies (taurus and indicus) (Hamilton *et al.* 2009; Mukherjee *et al.* 2013; Hughes *et al.* 2020). Also, *TSPY* is highly amplified in the domestic cat (∼100 copies) and shows considerable CNV between felids (Brashear *et al.* 2018). In contrast, no CNV between individuals or breeds has been observed for pig *TSPY* which has just 3 copies (Quach *et al.* 2015). On the other hand, our results across all study cohorts strongly suggest that there is only 1 copy of *HSFY* in horse MSY and not 3 copies as presented in eMSYv3 (Janečka *et al.* 2018). Single-copy *HSFY* in horses is more similar to the 2 copies in humans (Skaletsky *et al.* 2003; Vegesna *et al.* 2020) and 6 copies in gorilla (Tomaszkiewicz *et al.* 2016), but in stark contrast to cattle and pigs, where *HSFY* is massively amplified (Skinner *et al.* 2015; Hughes *et al.* 2020).

**Fig. 3.**
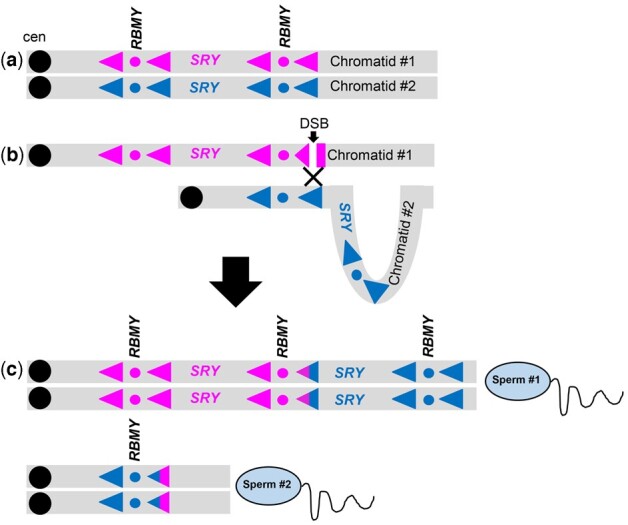
Schematic representation of the *SRY*-region in horse MSY proposing a mechanism for *SRY*/*RBMY* CNV during meiosis. a) Normal Y chromosome with 2 identical sister chromatids; highly similar directional repeats flanking *SRY* are denoted with arrowheads in pink and blue color to distinguish the same repeats in sister chromatids; the 2 copies of *RBMY* are indicated with pink and blue dots and the single-copy *SRY* in pink and blue font in the 2 sister chromatids, respectively. b) Proposed mechanism for the repair of double-stranded break (DBS) in 1 chromatid (arrow) by nonallelic homologous exchange (black cross) with an identical, but geographically distant repeat in the other chromatid as shown by sister chromatid misalignment and looping of chromatid #2. c) Outcomes of the nonallelic homologous exchange shown in (b). after sister chromatid separation in meiosis metaphase II, followed by DNA replication and sperm formation: sperm #1 carries a Y chromosome with 2 copies of *SRY* and 3 copies of *RBMY*, while sperm #2 has a single *RBMY* and no *SRY*. The latter leads to *SRY*-negative XY sex reversal. The idea of nonallelic homologous exchange between Y sister chromatids is adopted from Lange *et al.* (2009) and Teitz *et al.* (2018).

One of the most intriguing findings of this study was documenting 21 horses within the 209 normal male cohort with 2 or 3 copies of *SRY* (Table 3). Eighteen of these horses also had an extra copy of *RBMY* (rounded CN = 3), though 3 horses had a single *RBMY* instead of the normal 2. Not coincidentally, the cohort of 12 abnormal horses with *SRY*-negative XY DSD, had lost together with *SRY*, a copy of *RBMY* (Supplementary Table 7). Interrelationship of *SRY* and *RBMY* CNs is the consequence of the specific features of horse MSY structure where the single-copy *SRY* is embedded between almost 100% identical direct repeats, including 2 copies of *RBMY* (Janečka *et al.* 2018) (Fig. 3). Because MSY is not recombining, it maintains its genetic integrity by other mechanisms, of which one is homologous repair between sister chromatids. However, in structurally complex regions containing palindromes, inverted and direct repeats, exchange may happen between distant repeats located in different regions of the Y chromosome (nonallelic homologous repair), resulting in intrachromosomal structural rearrangements (Lange *et al.* 2009). For example, nonallelic homologous repair between chromatids in the horse *SRY*-region, may remove a segment with *SRY* and 1 copy of *RBMY* from 1 chromatid and add it to the other chromatid (Fig. 3). In meiosis, this will result in 2 different sperm: 1 with 2 copies of *SRY* and 3 copies of *RBMY*, another with a single *RBMY* and no *SRY* (Fig. 3). The latter will lead to *SRY*-negative 64, XY DSDs, also known as male-to-female sex reversal (Raudsepp *et al.* 2010). This scenario was initially proposed as a likely mechanism to explain the relatively high incidence of *SRY*-negative XY DSD in horses compared with other domestic species (Raudsepp *et al.* 2010). Structural complexity and likely instability of the *SRY*-region was further confirmed by the horse MSY reference assembly (Janečka *et al.* 2018) and is consistent with ddPCR results in *SRY*-negative 64, XY DSDs horses in this study (Supplementary Table 7). However, until the development of ddPCR assays, there have been no accurate tools to identify male horses with increased *SRY* and *RBMY* CN. It is certainly noteworthy that the 21 horses with more than 1 copy of *SRY* were in the normal male cohort and 7 were confirmed breeding stallions (Table 3), suggesting that elevated *SRY/RBMY* CNs have no negative phenotypic effect on fertility. It is though, puzzling that all males in this group were from small indigenous breeds (Table 3), while the “*other side of the same coin*”—*SRY*-negative 64, XY DSDs condition (Fig. 3), has been found in many common breeds (Raudsepp *et al.* 2010; Bugno-Poniewierska and Raudsepp 2021). At present, we do not have any plausible explanation why we did not detect any horses among common commercial breeds with elevated *SRY/RBMY* CN. We can only speculate that this may be associated with subtle phenotypic changes affecting human selection decisions in commercial breeds but have no importance in less-controlled indigenous horses.

To a very limited extent, we investigated MSY CNVs in related equids—5 Przewalski’s horses and 2 kulans—and observed lower overall CNV compared with the domestic horse. A notable difference from the domestic horse was that all 7 wild equids had a single copy of *SRY* and *RBMY* (Table 2 and Supplementary Table 1), suggesting that the structure of this region in these species may be different from horse MSY. Though, it is also possible that due to MSY sequence divergence, horse primers may not target all gene copies in other equids. In addition, the known low effective population size of these wild equids (Kaczensky *et al.* 2021) may facilitate elimination of new variants by genetic drift. Definite answers, however, need additional studies with more individuals and equid species. Otherwise, the successful use of all 8 ddPCR assays in Przewalski’s horse and 6 assays (except *TSPY* and *UBA1Y*) in kulan, suggests high degree of sequence conservation between these Y chromosomes.

Previously, sequence variation in the horse Y chromosome has been studied at single nucleotide level, which compared with other domestic species and wild equids, is outstandingly low (Wallner *et al.* 2003; Lindgren *et al.* 2004; Wallner *et al.* 2017). Nevertheless, the identified SNVs have allowed to determine HGs and HTs and trace the origin of patrilines and gain information about the relationships between horse breeds (Wallner *et al.* 2017; Felkel *et al.* 2019). Here, we generated information for another form of MSY variation—CNV of multicopy genes and showed that there is noorrelationn between Y CNs and HTs. For example, Estonian Native horses had similar CN patterns (Table 3) but separated into both Crown and non-Crown HGs based on SNVs. Likewise, the 2 Estonian Native horses (BP379 and BP380) with over 2 times higher than mean CN for *ETSTY2*, belonged to the most common Crown HG (Supplementary Table 1). Conversely, individuals from non-Crown HGs, did not necessarily stand out regarding their CN patterns, except the above discussed outlier—the Yakutian horse TR028.

Our observations are consistent with those in human and primates showing that SNV-based HGs do not cluster with CNV-based HGs (Ye *et al.* 2018; Vegesna *et al.* 2020). Also, similarly to primates, the studied 209-horse population showed much more diversity in MSY CNs compared with nucleotide diversity which defined a stable topology of 20 HTs (Fig. 2 and Supplementary Table 1). The same discordance between CNVs and SNVs was evident in successive male generations (Table 6)—CN showed variation, while HTs remained the same. This is because the sequence properties, molecular mechanisms, and evolutionary dynamics underlying CNVs and SNVs are substantially different. The majority of SNVs that determine HGs reside in MSY single-copy nongenic or intronic sequences and are mainly influenced by mutations which occur at a rate as low as 1.69 × 10^−8^ mutations/site/generation (Felkel *et al.* 2019). This is clearly different from CNVs of functional genes in structurally complex ampliconic sequences which are prone for structural rearrangements by inter- and intrachromatid exchanges and gene conversion (Lange *et al.* 2009) (see Fig. 3). Also, our findings of transgenerational CN changes, as well as of CNVs between cloned horses suggest that these structural rearrangements can be of both meiotic (germline) and mitotic (somatic) origin.

In humans where Y chromosome research is currently the most advanced, analysis of high-throughput sequencing data from over 1,200 males has allowed to accurately detect CN of MSY ampliconic genes in each individual, but also to determine the ancestral reference CN for each gene (Teitz *et al.* 2018). It appears that even though there is CNV between individuals, the reference (ancestral) CN of each ampliconic gene is rigorously maintained, indicative of mutation-selection balance. The presence of selective constraints on amplicon CN in human Y chromosome, suggests that MSY CNVs have phenotypic effects, most likely on spermatogenesis (Teitz *et al.* 2018). It is too early to comment about whether and how this may apply to horse MSY CNVs, but the idea is important regarding stallion fertility and worth pursuing in future research. For example, even though in this study we determined the mean baseline CN for 8 MSY genes (Table 2), a much larger and more diverse equine population is needed to find out whether the determined baseline CN is also the ancestral condition. The lack of such information, combined with the overall limited structural and functional knowledge about the horse MSY ampliconic region, also sets limits to interpret CN analysis results in cryptorchid and infertile/subfertile males. The observed lower CN of *TSPY* and *ETSTY2* in cryptorchid AQHs (Table 5) left only questions. On the one hand, the same 2 genes were most variable also in the normal cohort (Table 2); thus, it is possible that the small sample size and the known heterogeneity of the cryptorchid phenotype (Amann and Veeramachaneni 2007) may have skewed the statistics. On the other hand, if the association is true, we have no knowledge about the functions of the equine-specific transcript *ETSTY2* or the horse *TSPY* gene. CNV of the latter has been associated with subfertility phenotypes in men [reviewed by Rogers (2021)] and lower semen quality in bulls (Mukherjee *et al.* 2015) but not with cryptorchidism. The fact that we did not detect any significant CNV among subfertile/infertile stallions is likely the consequence of too many diverse phenotypes, each with very small sample sizes. Furthermore, most MSY multicopy and ampliconic genes have not yet been functionally annotated in horses or any other domestic species, which greatly limits the understanding of their role in stallion biology.

### Conclusions and future directions

We showed that ddPCR is a reliable approach for CNV analysis of horse MSY multicopy genes and provides a more accurate CN evaluation compared with the current assembly of the ampliconic region in MSY reference eMSYv3 (Janečka *et al.* 2018). Gene CN analysis in a large multibreed population of normal male horses showed that most multicopy MSY genes show CNV between individuals, breeds, but also in successive male generations and horses produced by SCNT. This suggests that MSY gene CNVs are caused by both meiotic and mitotic events and are mechanistically different from SNVs that are rare and determine Y chromosome HTs. Therefore, MSY CNV patterns are not correlated with HGs and HTs. Further studies are needed to determine selective constraints over horse MSY gene CN and how this relates to equine male development and fertility. For this and for the inclusion of the ampliconic genes that were missed in this study, the sequence assembly of the horse MSY ampliconic region must be improved. This will require a combined use of cutting-edge platforms for the assembly of complex genomic regions such as PacBio single-molecule, high-fidelity, long-read sequencing (Vollger *et al.* 2020), and Bionano optical mapping (Bocklandt *et al.* 2019). An improved MSY ampliconic assembly is also the prerequisite for functional annotation of these genes to determine their role in stallion reproduction and male biology.

## Supplementary Material

jkac278_Supplemental_Material_Legends

jkac278_Supplemental_Table_S1

jkac278_Supplemental_Table_S2-S8

## Data Availability

The authors state that all data necessary for confirming the conclusions presented in the article are represented fully within the article and supplementary material. Supplemental material is available at G3 online.
