# Supplementary material for: Copy number variation of horse Y chromosome genes in normal equine populations and in horses with abnormal sex development and subfertility: relationship of copy number variations with Y haplogroups
Source: G3 (Bethesda). 2022 Oct 13;12(12):jkac278. doi: 10.1093/g3journal/jkac278 (PMC9713435; doi:10.1093/g3journal/jkac278)
Supplement: jkac278_Supplemental_Material_Legends [file jkac278_supplemental_material_legends.docx]

**Supplemental material**

**Table S1.1 - S1.8:** Detailed information for all 289 horses (horse ID, HG, HT, sample type, karyotype, *SRY* PCR, phenotype, and breed) involved in this study, together with composite information for CN analysis of the 8 MSY genes by ddPCR (mean CN, SD, reference gene used, number of ddPCR experiments, CNs of each replicate experiment); individuals in red font are missing CNs for some genes (DNA sample finished).

**Table S2:**  Summary data of different statistical analyses for CN variation across horse breeds, HGs, and between normal and cryptorchid horses.

**Table S3:** Droplet digital PCR assays for the Y chromosome genes analyzed in this study; the location of the assay in the gene, and the expected number of copies is based on eMSYv3 reference assembly (Janecka et al., 2018).

**Table S4:** Detailed information for the 30 MSY SNVs that determined individual HGs and HTs.

**Table S5:** Table S5: MSY HGs, HTs and corresponding SNV genotypes of 216 normal male horses and equids.

**Table S6:** Individual copy numbers of the 7 MSY multi-copy genes and *SRY* of cryptorchid Quarter Horses and normal Quarter Horse controls; numbers in blue font denote the largest CN value and numbers in pink font denote the lowest CN value.

**Table S7:** Individual copy numbers of the 7 MSY multi-copy genes and *SRY* in horses with confirmed 64,XY disorders of sex development (XY DSDs), together with the CN range for respective breed in normal cohort; numbers in blue font denote the largest CN value and numbers in pink font denote the lowest CN value; (-) shows that CNs were not generated due to missing content.

**Table S8:** Individual copy numbers of the 7 MSY multi-copy genes and *SRY* in subfertile/infertile males, cloned horses and the normal male SCNT donor, together with the CN range for respective breed in normal cohort; numbers in blue font denote the largest CN value and numbers in pink font denote the lowest CN value.
